# Validation of deep-learning accelerated quantitative susceptibility mapping for deep brain nuclei

**DOI:** 10.3389/fnins.2025.1522227

**Published:** 2025-01-22

**Authors:** Ying Zhou, Lingyun Liu, Shan Xu, Yongquan Ye, Ruiting Zhang, Minming Zhang, Jianzhong Sun, Peiyu Huang

**Affiliations:** ^1^Taizhou Central Hospital (Taizhou University Hospital), Taizhou, China; ^2^The Second Affiliated Hospital, Zhejiang University School of Medicine, Hangzhou, China; ^3^United Imaging, Houston, TX, United States

**Keywords:** quantitative susceptibility mapping, acceleration, brain nuclei, deep learning, parallel imaging

## Abstract

**Purpose:**

To test the feasibility and consistency of a deep-learning (DL) accelerated QSM method for deep brain nuclei evaluation.

**Methods:**

Participants were scanned with both parallel imaging (PI)-QSM and DL-QSM methods. The PI- and DL-QSM scans had identical imaging parameters other than acceleration factors (AF). The DL-QSM employed Poisson disk style under-sampling scheme and a previously developed cascaded CNN based reconstruction model, with acquisition time of 4:35, 3:15, and 2:11 for AF of 3, 4, and 5, respectively. For PI-QSM acquisition, the AF was 2 and the acquisition time was 6:46. The overall image similarity was assessed between PI- and DL-QSM images using the structural similarity index (SSIM) and peak signal-to-noise ratio (PSNR). QSM values from 7 deep brain nuclei were extracted and agreements between images with different Afs were assessed. Finally, the correlations between age and QSM values in the selected deep brain nuclei were evaluated.

**Results:**

59 participants were recruited. Compared to PI-QSM images, the mean SSIM of DL images were 0.87, 0.86, and 0.85 for AF of 3, 4, and 5. The mean PSNR were 44.56, 44.53, and 44.23. Susceptibility values from DL-QSM were highly consistent with routine PI-QSM images, with differences of less than 5% at the group level. Furthermore, the associations between age and QSM values could be consistently revealed.

**Conclusion:**

DL-QSM could be used for measuring susceptibility values of deep brain nucleus. An AF up to 5 did not significantly impact the correlation between age and susceptibility in deep brain nuclei.

## 1 Introduction

Various substances in the human body, such as iron calcium, and lipids, can alter magnetic susceptibility (Deistung et al., 2017). Abnormal changes in their content are often associated with the progression of specific diseases. In recent years, Quantitative susceptibility mapping (QSM) has been widely applied in clinical disease research, including neurodegenerative diseases (Shahmaei et al., 2019; Guan et al., 2017), vascular diseases (Probst et al., 2021; Hong et al., 2023), neuroinflammation (Chen et al., 2014), and traumatic brain injury (Chary et al., 2021). For example, in Parkinson’s disease, QSM values in the substantia nigra can be used to differentiate between patients and controls (Shahmaei et al., 2019; Guan et al., 2017), and QSM images can assist in the preoperative localization of deep brain nuclei (Cong et al., 2020). In Alzheimer’s disease, QSM can reflect pathological iron deposition associated with amyloid protein (Gong et al., 2019).

In conventional and clinical research applications, susceptibility-weighted imaging mainly uses three-dimensional GRE sequences. To achieve a strong susceptibility effect, a relatively long echo time is required to establish phase contrast (usually >30 ms for a 3 T MRI scanner) in QSM imaging, and multiple echoes are typically acquired to compute susceptibility (Shahmaei et al., 2019; Guan et al., 2017; QSM Consensus Organization Committee et al., 2024). For visualizing fine anatomical structures (such as small venules and cortical structures) and small lesions (such as microbleeds) (Rotta et al., 2021), a high spatial resolution is also required (Shen et al., 2020). A recent consensus paper suggests using isotropic acquisition protocols to avoid partial volume effect (QSM Consensus Organization Committee et al., 2024). The combination of these various demands can significantly extend acquisition times, thereby reducing patient compliance.

In recent years, deep learning (DL) methods have gained widespread attention and application in the field of magnetic resonance imaging (MRI), being utilized in various directions such as accelerating image acquisition, reducing gadolinium dose (Gong et al., 2018), and improving image resolution (Avanzo et al., 2020; Johnson et al., 2023; Almansour et al., 2023). In terms of accelerating MRI acquisition, DL methods are primarily used to recover k-space information from undersampled k-space data or to directly reconstruct images (Han et al., 2020; Wang et al., 2021). They can also enhance image quality based on low-quality images reconstructed from undersampled data (Bash et al., 2021). Previously, Gao (Gao et al., 2021) et al. trained a Deep Complex Residual Network (DCRNet) using 7 T MRI data. They tested the model on several retrospectively under-sampled datasets and one prospectively under-sampled dataset, demonstrating substantially reduced artifacts and blurring compared to two iterative methods and one deep learning method. Similarly, Zhang (Zhang et al., 2023) et al. developed a framework, Learned Acquisition and Reconstruction Optimization (LARO), designed to accelerate multi-echo GRE pulse sequences for QSM. The authors optimized the k-space sampling pattern and employed a recurrent temporal feature fusion model to capture signal redundancies along echo times. Their methods were tested on prospectively under-sampled k-space datasets from 10 healthy subjects, achieving an acceleration factor of 8 while maintaining QSM quality. While these studies demonstrated the feasibility of applying DL methods to QSM acquisition, they primarily focused on methodology development. The generalizability and stability of DL-based QSM remain untested in larger sample sizes.

In this study, we aimed to test whether a DL-based reconstruction method could be used for accelerating QSM acquisition without significant impact on the measurement of susceptibility in deep brain nuclei. Specifically, we investigated the overall image similarity between PI and DL-QSM images using the structural similarity (SSIM) and peak signal-to-noise ratio (PSNR) indices. We tested the correlation and differences between different sets of QSM images. At last, we investigated whether the acceleration would affect the correlation analyses between age and nuclei QSM values.

## 2 Methods

### 2.1 Participants

The research protocol has been approved by the ethics committee of the Second Affiliated Hospital, Zhejiang University School of Medicine. We recruited healthy participants from nearby communities through online advertising. The inclusion criteria were (1) between age 18 ~ 70; (2) without history of neuropsychiatric diseases or severe systematic diseases that could affect the brain; and (3) passing the routine MR safety screening. The exclusion criteria were (1) unable to hold still in the scanner; (2) accidental findings of brain occupying lesions. All participants signed informed consent before enrollment. A total of 60 participants were enrolled, but one participant was excluded due to head motion and data corruption. For all participants, demographic and clinical information were recorded.

### 2.2 DL-based acceleration method

The DL-QSM method uses a cascaded CNN model, namely ReconNet3D (Ye et al., 2023), to perform k-space to image reconstruction with single-channel input and single-channel output capacity, i.e., data from each channel were independently reconstructed (Ye et al., 2023). The model has five 3D convolutional blocks and data consistency layers, and each convolutional block contains five convolutional layers. It was trained on 42 multi-flip-angle and multi-echo GRE brain scans (a total of 662 volumes of 3D data), acquired with fully sampled scans. The ground truth data were then retrospectively under-sampled using 3X and 5X Poisson-disk schemes (Akasaka et al., 2016) in both the phase-encoding and slice directions. These under-sampled data were used as inputs for model training. Additionally, another 5 multi-echo GRE brain scans (75 volumes of 3D echo data) were used for testing. For 3X and 5X under-sampled data, the testing results showed good agreement with the ground truth. The trained model was then tested with scans from both phantoms and human volunteers and demonstrated good reconstruction accuracy (Ye et al., 2023).

### 2.3 Imaging acquisition

MRI examinations were performed on a 3 T scanner (uMR790, United Imaging Healthcare, Shanghai, China) with a 32-channel head coil. QSM images were acquired using a traditional 3D multi-gradient-echo sequence equipped with both parallel imaging (PI) and DL method supporting 4 ~ 6 fold acceleration. The parameters were: TR = 30.2 ms, first TE = 3.3 ms, last TE = 25.0 ms, number of echoes = 8, echo spacing = 3.1 ms, flip angle = 15°, voxel size = 0.8 mm * 0.8 mm * 2 mm, covering the whole brain. All parameters were identical for both QSM scans except for acceleration factors. For the DL-QSM acquisition, the k-space was prospectively under-sampled using the Poisson disk scheme (Akasaka et al., 2016). The scan time of PI image was 6:46 with AF of 2, while the scan time of the DL-QSM scan was 4:35, 3:15, and 2:11 with AF of 3, 4, and 5, respectively. A 3D T1-weighted image was also acquired from each participant for registration and segmentation purposes. The parameters were: TR = 6.9 ms, TE = 2.9 ms, flip angle = 9°, TI = 1,000 ms, field of view (FOV) = 256 × 240 mm^2^, voxel size = 1 × 1 × 1 mm^3^, 208 sagittal slices.

### 2.4 Image processing

QSM reconstruction was performed by the built-in QSM reconstruction pipeline on the scanner (QSM Consensus Organization Committee et al., 2024; Ye et al., 2019). Specifically, the preliminary extraction of the field map employed the recently proposed Multi-Dimensional Integration (MDI) algorithm (Ye et al., 2022), which utilizes the signal channel dimensions of array coils to remove various unknown unrelated phases (such as the inherent phase of coil sensitivity) while obtaining the original field map containing magnetic susceptibility phase and background field phase. After obtaining the original field map, it underwent an exact phase unwrapping using the SPUN algorithm (Ye et al., 2019) based on region-growing with prioritized seed point screening, and then low-frequency background field components were removed using the vSHARP method (Wu et al., 2012), thereby obtaining a relative dipole field (RDF). Finally, an iterative L1-Norm based optimization calculation procedure was performed on the RDF field map to generate the QSM map. Dynamic Bayesian terms were incorporated during the optimization process to reduce artifacts and improve calculation accuracy and efficiency (Ye et al., 2019; Bilgic et al., 2014). To avoid motion-induced displacement of the head, all DL images were first co-registered to the PI images, using the FLIRT tool in the FMRIB Software Library (FSL, 6.0.1^1^).

### 2.5 Image similarity assessment

To quantitatively assess the similarity between the PI and DL images, we calculated structural similarity index (SSIM) and peak signal-to-noise ratio (PSNR) using Matlab (R2019a). Both indices have been widely used to assess image reconstruction qualities in medical imaging (Knoll et al., 2020). SSIM measures nonlocal structural similarity, which considers luminance, contrast, and structure. SSIM ranges from 0 to 1, with a large value representing better consistency. The PSNR measures voxel-wise differences between the images. Typically, a PSNR higher than 40 dB suggests excellent image quality.

### 2.6 Extraction of susceptibility values

**Figure 1:**
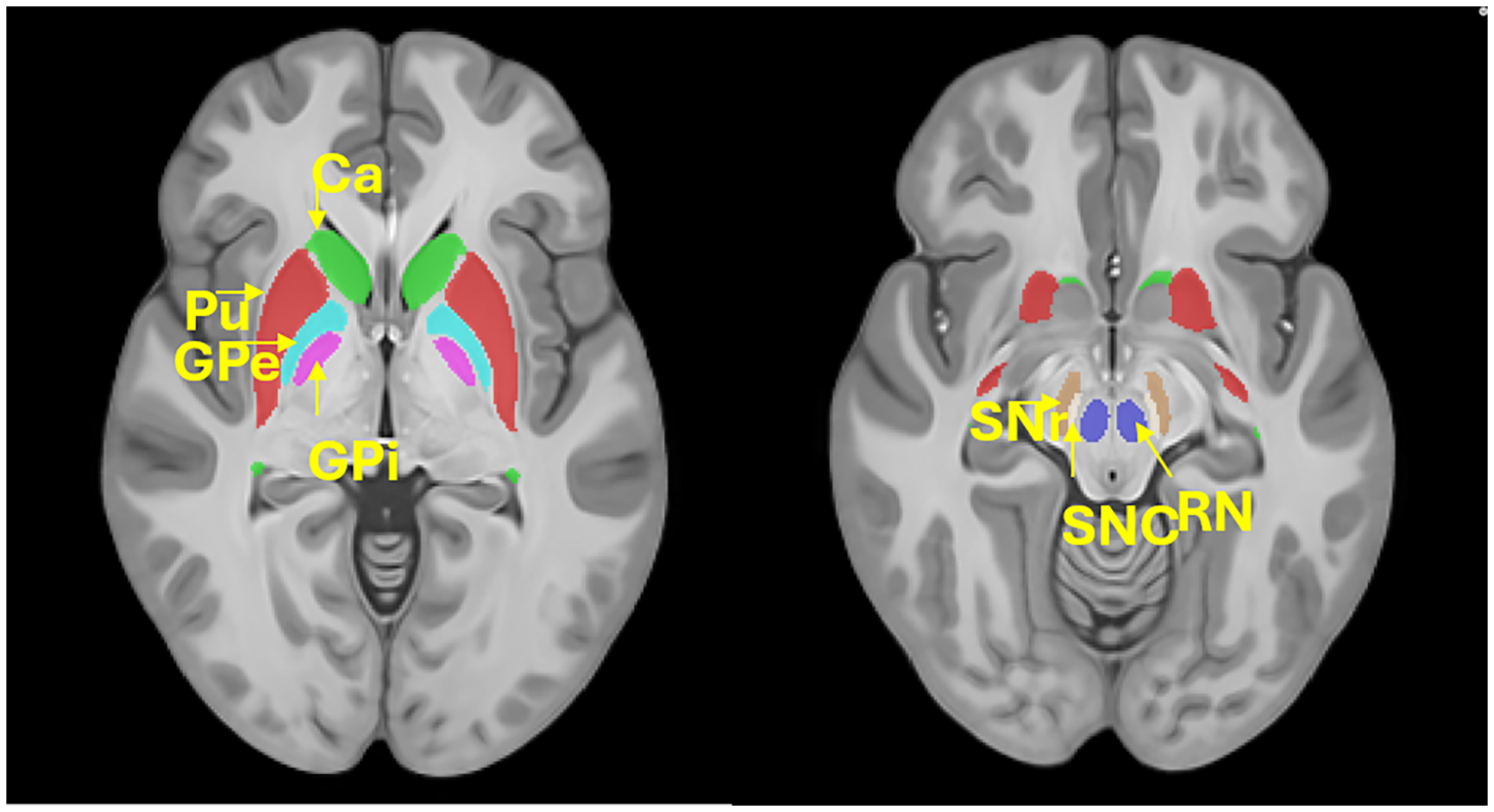
Location of the deep brain structures. Putamen, Pu; Caudate, Ca; external globus pallidus, GPe; internal globus pallidus, GPi; Red nucleus, RN; substantia nigra pars compacta, SNc; nigra pars reticulata, SNr.

We used SEPIA,^2^ a versatile tool for QSM image processing (Chan and Marques, 2021), to extract susceptibility values from deep brain nuclei. Specifically, a brain structural atlas (Pauli et al., 2018) was registered to each participant’s magnitude image using ANTs registration.^3^ Both linear and non-linear registrations were used to achieve accurate alignment. Registration accuracy was visually checked. Then the registered atlas was used to extract QSM values from seven large and important deep brain structures (Figure 1, Putamen, Pu; Caudate, Ca; external globus pallidus, GPe; internal globus pallidus, GPi; Red nucleus, RN; substantia nigra pars compacta, SNc; nigra pars reticulata, SNr).

### 2.7 Statistical analyses

We first examined the correlation between QSM values derived from different sets of images using Pearson’s correlation. Then we compared the difference between PI- and DL-QSM images using paired t-tests. The Bland–Altman plot was used to show difference between each pair of PI-DL values. Finally, we tested the association between age and susceptibility values in each deep brain nucleus to investigate whether acquisition acceleration might affect the results.

## 3 Results

A total of 59 participants were enrolled in this study (Table 1, mean age: 44.0 y/o; range: 18–75 y/o; male/female: 22/37). Using PI-QSM images as reference, the mean SSIM of DL-QSM images were 0.87 (min-max: 0.82–0.89), 0.86 (0.77–0.89), and 0.85 (0.81–0.89) for acceleration factors of 3, 4, and 5, and respective mean PSNR were 44.56 (37.31–52.12), 44.53 (37.42–53.10), and 44.23 (37.35–52.91).

**Table 1:** Demographic information.

	*N* = 59
Age, year, mean ± SD	44.0 ± 15.4
Female, *N* (percent)	37 (62.7%)
Education	11.7 ± 3.0
Body mass index	22.8 ± 3.4
Hypertension, *N* (percent)	5 (8.5%)
Diabetes, *N* (percent)	0 (0%)
Hyperlipidemia, *N* (percent)	2 (3.4%)
Smoking, *N* (percent)	10 (16.9%)

Raw data were presented as mean (± standard deviation [SD]) or number (percentage, %) in tables unless otherwise noted.

**Figure 2:**
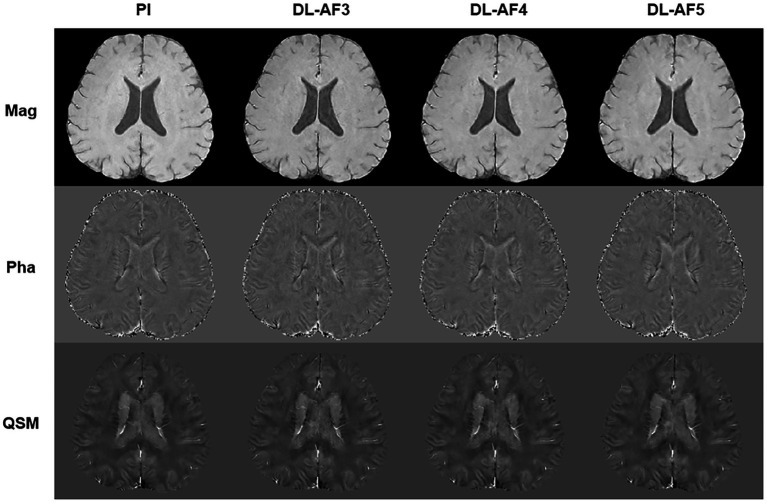
Demonstration of PI and DL images. AF, Acceleration factor; Mag, magnitude image; Pha, phase image; QSM, quantitative susceptibility mapping.

**Figure 3:**
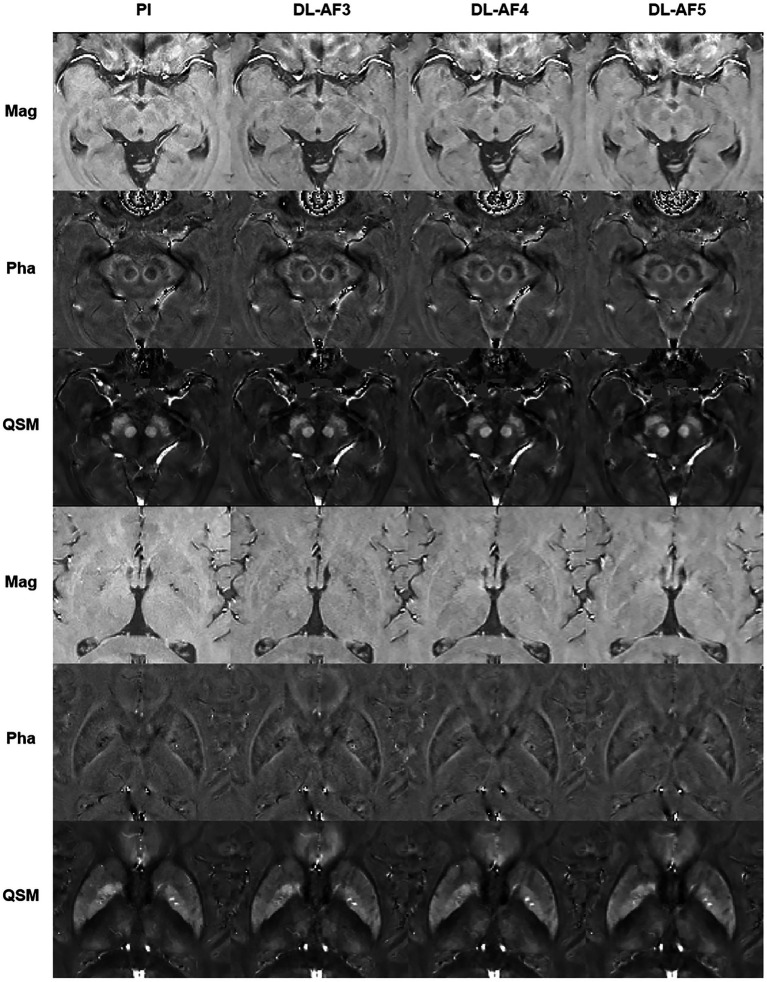
Demonstration of the imaging details in PI and DL images. AF, Acceleration factor; Mag, magnitude image; Pha, phase image; QSM, quantitative susceptibility mapping.

As shown in Figure 2, under different acceleration factors, DL-QSM exhibits a similar overall appearance compared to PI images. Figure 3 shows imaging details in the basal ganglia and brainstem regions. In general, the shape and local patterns of deep brain nuclei were mostly preserved, with a tendency toward smoother images under higher acceleration conditions.

As shown in Table 2, the susceptibility values of the seven deep brain structures in each DL-QSM group have a very high correlation with the PI-QSM group (*r* > 0.95, *p* < 0.001). In the PI-QSM and DL-QSM-AF3 groups, the GPe showed the highest correlation (*r* = 0.994, *p* < 0.001), while the RN showed the lowest correlation (*r* = 0.963, *p* < 0.001). In the PI-QSM and DL-QSM-AF4 groups, the GPe also showed the highest correlation (*r* = 0.993, *p* < 0.001), while the SNr showed the lowest correlation (*r* = 0.950, *p* < 0.001). In the PI-QSM and DL-QSM-AF5 groups, the GPe again showed the highest correlation (*r* = 0.993, *p* < 0.001), while the Ca showed the lowest correlation (*r* = 0.950, *p* < 0.001). Overall, among the PI-QSM group and all DL-QSM groups, the GPe showed the highest susceptibility value correlation.

**Table 2:** Associations between susceptibility values derived from PI and DL images.

	QSM_PI - DL_AF3		QSM_PI - DL_AF4		QSM_PI - DL_AF5	
	*r*	*p*	*r*	*p*	*r*	*p*
Pu	0.991	<0.001	0.990	<0.001	0.987	<0.001
Ca	0.968	<0.001	0.965	<0.001	0.961	<0.001
GPe	0.994	<0.001	0.993	<0.001	0.993	<0.001
GPi	0.980	<0.001	0.988	<0.001	0.978	<0.001
SNc	0.973	<0.001	0.956	<0.001	0.973	<0.001
SNr	0.972	<0.001	0.950	<0.001	0.975	<0.001
RN	0.963	<0.001	0.954	<0.001	0.963	<0.001

QSM, Quantitative susceptibility mapping; PI, parallel imaging; DL, deep-learning; AF, acceleration factor; Pu, Putamen; Ca, Caudate; GPe, external globus pallidus; GPi, internal globus pallidus; SNc, substantia nigra pars compacta; SNr, nigra pars reticulata; RN, Red nucleus.

On the group level (Table 3), the region with the greatest susceptibility difference between the PI-QSM and DL-QSM-AF3 images is the SNr, with a mean difference of 4.19%; followed by the Ca, with a mean difference of 3.31%. The greatest difference between PI-QSM and DL-QSM-AF4 is in the Ca, at 2.98%; while the greatest difference between PI-QSM and DL-QSM-AF5 is in the Pu, at 3.15%. There was no systematic bias between the values from different image groups.

**Table 3:** Pair-wise comparison between PI and DL images.

	QSM_PI	QSM_DL_AF3		QSM_DL_AF4		QSM_DL_AF5	
	Mean	Mean	Diff	Mean	Diff	Mean	Diff
Pu	30.8 ± 12.9	30.7 ± 13.1	−0.56%	31.2 ± 13.4	1.24%	31.8 ± 13.5	**3.15%**
Ca	29.6 ± 6.3	28.6 ± 6.3	**−3.31%**	28.7 ± 6.5	**−2.98%**	29.4 ± 6.7	−0.82%
GPe	120.2 ± 34.3	118.3 ± 35.4	**−1.58%**	119.6 ± 35.8	−0.45%	120.3 ± 35.1	0.11%
GPi	92.3 ± 26.0	90.4 ± 24.8	**−2.06%**	92.0 ± 24.8	−0.32%	94.4 ± 28.0	**2.33%**
SNc	84.6 ± 27.2	83.6 ± 25.8	−1.18%	85.3 ± 26.7	0.77%	86.7 ± 27.7	**2.49%**
SNr	99.9 ± 25.4	95.7 ± 24.8	**−4.19%**	100.3 ± 27.2	0.40%	102.6 ± 28.3	**2.71%**
RN	65.1 ± 23.1	64.3 ± 23.7	−1.15%	66.1 ± 23.7	1.57%	65.1 ± 25.0	0.07%

QSM, Quantitative susceptibility mapping; PI, parallel imaging; DL, deep-learning; AF, acceleration factor; Pu, Putamen; Ca, Caudate; GPe, external globus pallidus; GPi, internal globus pallidus; SNc, substantia nigra pars compacta; SNr, nigra pars reticulata; RN, Red nucleus. Bold represent significant correlation.

**Figure 4:**
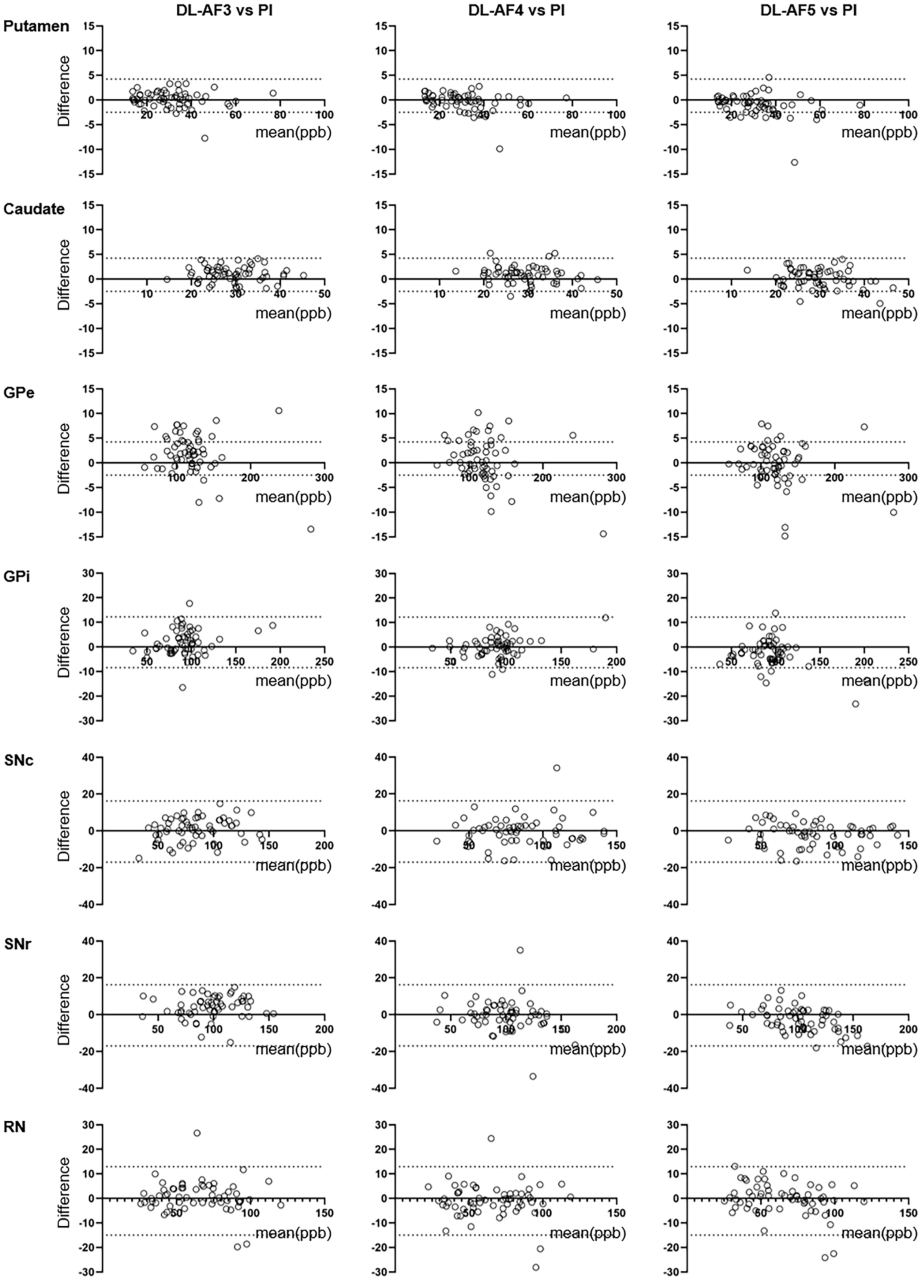
Bland–Altman plots showing consistency between QSM values extract from PI and DL images. The consistency was higher for larger nuclei (e.g., putamen, caudate) compared to smaller nuclei (e.g., GPi, SNc). GPe, external globus pallidus; GPi, internal globus pallidus; RN, Red nucleus; SNc, substantia nigra pars compacta; SNr, substantia nigra pars reticulata.

**Figure 5:**
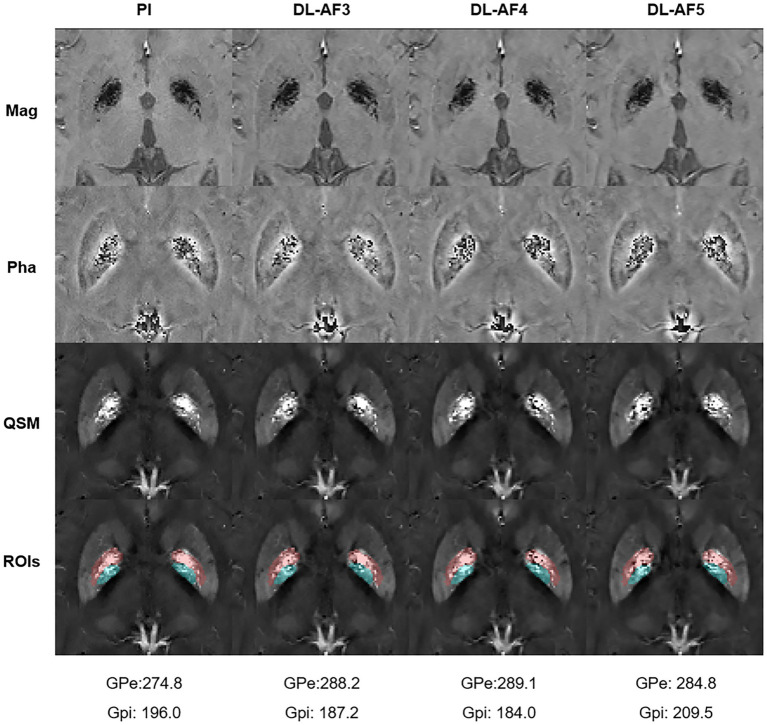
One participant showing relatively large differences in GPi and GPe between PI and DL images. Please notice the subtle differences within these regions (Pink: GPe; Cyan: GPi). ROIs, Regions of interest.

As shown on the Bland–Altman plot (Figure 4), the differences between PI and DL-QSM images distributed evenly across different susceptibility levels. For putamen and caudate, differences between the PI and DL images were mostly less than 5 ppb. For other nuclei, the difference was higher, reaching over 10 ppb. When looking into the source of these discrepancies, we noticed the influence of smoothness on local patterns in the QSM images (Figure 5). In some cases, mild head motion and imaging artifacts might also contribute to the differences (Table 4).

**Table 4:** Correlation between susceptibility values and age in different sets of images.

	QSM_PI		QSM_DL_AF3		QSM_DL_AF4		QSM_DL_AF5	
	*r*	*p*	*r*	*p*	*r*	*p*	*r*	*p*
Pu	0.631	**<0.001**	0.644	**<0.001**	0.653	**<0.001**	0.658	**<0.001**
Ca	0.273	**0.036**	0.284	**0.029**	0.224	0.088	0.247	0.060
GPe	0.182	0.167	0.180	0.171	0.170	0.198	0.170	0.198
GPi	0.002	0.986	0.025	0.851	−0.020	0.878	0.004	0.976
SNc	0.328	**0.011**	0.302	**0.020**	0.316	**0.015**	0.366	**0.004**
SNr	0.093	0.485	0.026	0.843	0.032	0.812	0.080	0.546
RN	0.504	**<0.001**	0.498	**<0.001**	0.517	**0.012**	0.502	**<0.001**

QSM, Quantitative susceptibility mapping; PI, parallel imaging; DL, deep-learning; AF, acceleration factor; Pu, Putamen; Ca, Caudate; GPe, external globus pallidus; GPi, internal globus pallidus; SNc, substantia nigra pars compacta; SNr, nigra pars reticulata; RN, Red nucleus. Bold represent significant correlation.

In the PI-QSM images, the susceptibility values of the Pu, Ca, SNc, and RN are positively correlated with age and have statistical significance, with the Pu showing the strongest correlation with age (*r* = 0.631, *p* < 0.001). In the other three DL-QSM image groups, the susceptibility values of the Pu, SNc, and RN also show significant positive correlations with age, with the Pu showing the strongest correlation with age (AF-3: *r* = 0.644, *p* < 0.001; AF-4: *r* = 0.653, *p* < 0.001; AF-5: *r* = 0.658, *p* < 0.001). The Ca shows a significant correlation with age in the DL-QSM-AF3 images (*r* = 0.284, *p* = 0.029) and a marginally significant positive correlation with age in the DL-QSM-AF4 (*r* = 0.224, *p* = 0.088) and DL-QSM-AF5 images (*r* = 0.247, *p* = 0.060).

## 4 Discussion

This study showed that DL-QSM images with acceleration factors of 3, 4, and 5 had good similarity to PI-QSM images, with high correlation in the measured susceptibility values of nuclei (*r* > 0.95). When the acceleration factor was 5, the required scanning time was only one-third of the PI-QSM acquisition time, greatly improving scanning efficiency. Although there were differences in some nuclei between different QSM images, these differences were usually less than 5% on the group level. Such small variations do not significantly affect the correlation analyses between age and QSM values.

Under the condition of 5-fold acceleration, we did not observe significant differences in the overall image appearance and nuclei structures. It should be noted that as the k-space signal sampling decreased, the images appeared slightly smoother, which might lead to slight confusion of signals in adjacent nuclei or white matter areas. The smoothing might have affected the voxel values at the nuclei boundaries and causing fluctuations in the susceptibility values of some nuclei regions. However, the differences in susceptibility values of these nuclei are general small on the group level.

On the individual level, larger differences might occur in some participants. Such variations could be due to more complex reasons besides the abovementioned smoothing effect. Head motion between scans could change the angle between the acquisition plane and the main magnetic field direction, which might alter the susceptibility effect of the dipoles. Small head motion during each QSM acquisition could cause artifacts and affect QSM estimation. There might be other factors such as magnetic field shimming, temperature changes, and various imaging artifacts. One study scanned phantoms containing five different concentrations of gadolinium solutions using 12 clinical and 3 preclinical scanners (Deh et al., 2019). At the level of 0.26 ppm, the standard deviation of the measurements was 32 ppb. In a replication study that scanned 14 participant four times using an identical scanner and the same protocol, the standard deviation of QSM values in deep brain nuclei across different imaging sessions can reach 19 ppb (Santin et al., 2017). In our study, differences between PI and DL images were at a comparable level (mostly less than 20 ppb), suggesting the influence of undersampling and DL reconstruction could be tolerated.

The basal ganglia and brainstem nuclei are key regions of the brain responsible for processing motor control, emotions, cognition, and various other functions (Groenewegen, 2003; Seger, 2006). Due to metabolic changes, vascular damages, and other factors, significant iron deposition can occur in these nuclei during brain aging (Ashraf et al., 2018). Some researchers have used QSM to study normal populations, showing that the susceptibility values of multiple nuclei in normal people tend to gradually increase with age (Acosta-Cabronero et al., 2016; Li et al., 2023). Consistent with previous results (Acosta-Cabronero et al., 2016), our study also confirmed that the Pu region shows the most significant increase in susceptibility, followed by the RN, SNc, and Ca regions. Furthermore, the correlations between age and DL-QSM values were similar to those with PI-QSM values, suggesting that the acceleration did not induce unexpected impacts to the analyses.

Although the current study preliminarily demonstrates the potential of DL-QSM sequences in magnetic resonance imaging, the robustness and general applicability of the conclusions need to be validated in larger-scale cohort studies due to the limited sample size. Particularly, expanding the sample size for different groups of patients with neurological disorders will help comprehensively evaluate the stability and diagnostic accuracy of DL-QSM sequences under various pathological conditions. Secondly, under the current experimental conditions, using an MR scanner equipped with an NVIDIA GeForce RTX 2080 Ti graphics card, image reconstruction takes several minutes to generate QSM images. Upgrading to newer, more powerful graphics cards could significantly improve image processing speed. Thirdly, while using fully sampled data as a reference is ideal, the long acquisition time may result in a high failure rate in clinical settings. Therefore, we used parallel imaging data as the reference. Since the model performance has already been assessed, and our primary goal was to evaluate the practicality of this method, comparing the image quality of DL-QSM with clinically established methods is considered acceptable. Furthermore, three-dimensional high-resolution QSM images have important value in preoperative nuclei localization, our future research will further investigate these related topics.

In summary, DL-QSM could be used for measuring susceptibility values of deep brain nuclei. An AF up to 5 did not significantly impact the accuracy of QSM results, nor the correlation between age and susceptibility in deep brain nuclei.

## Data Availability

The datasets presented in this article are not readily available because the data currently being applied has not been obtained from publicly available databases. If you wish to use this data, it must be reviewed by our committee. Requests to access the datasets should be directed to huangpy@zju.edu.cn.

## Footnotes

^1^ https://fsl.fmrib.ox.ac.uk/fsl/fslwiki

^2^ https://github.com/kschan0214/sepia

^3^ https://stnava.github.io/ANTs/
